# Supplementary figures and images for: Characterization, Expression, and Interaction Analyses of OsMORF Gene Family in Rice
Source: Genes (Basel). 2019 Sep 10;10(9):694. doi: 10.3390/genes10090694 (PMC6770982; doi:10.3390/genes10090694)

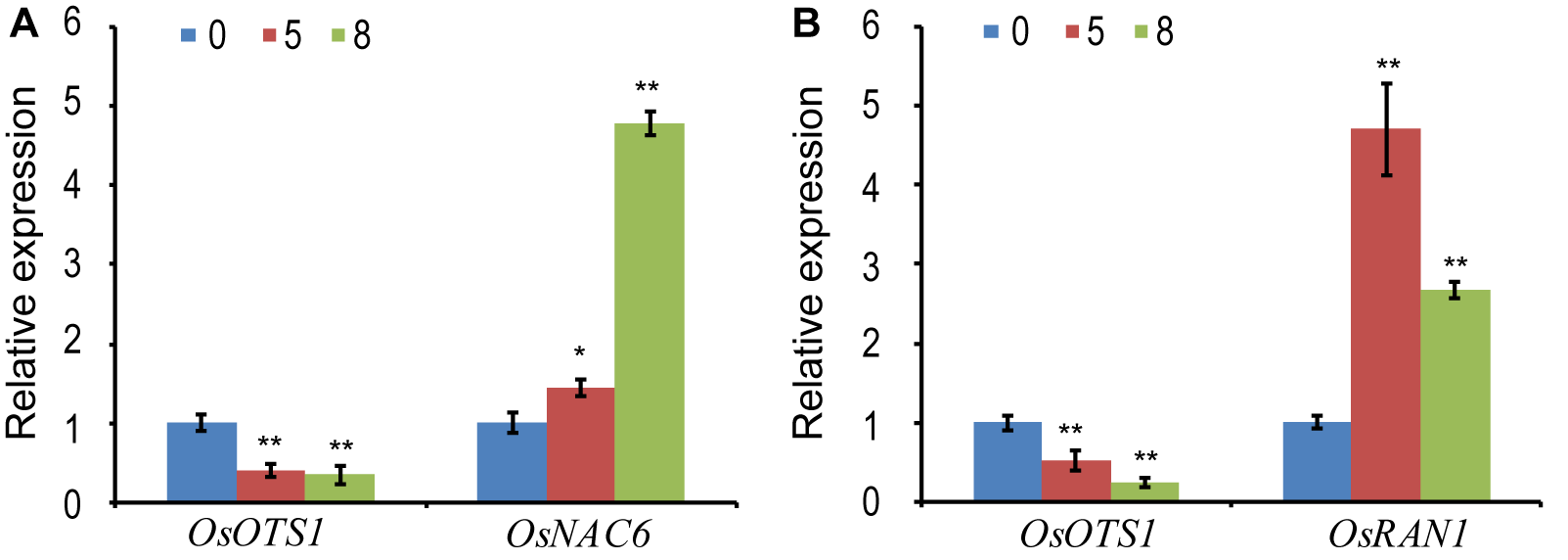

Supplement: Supplementary file 1 [file genes-10-00694-s001.zip › genes-576238- supplemantary/supplementary materials/FigureS1.tif]

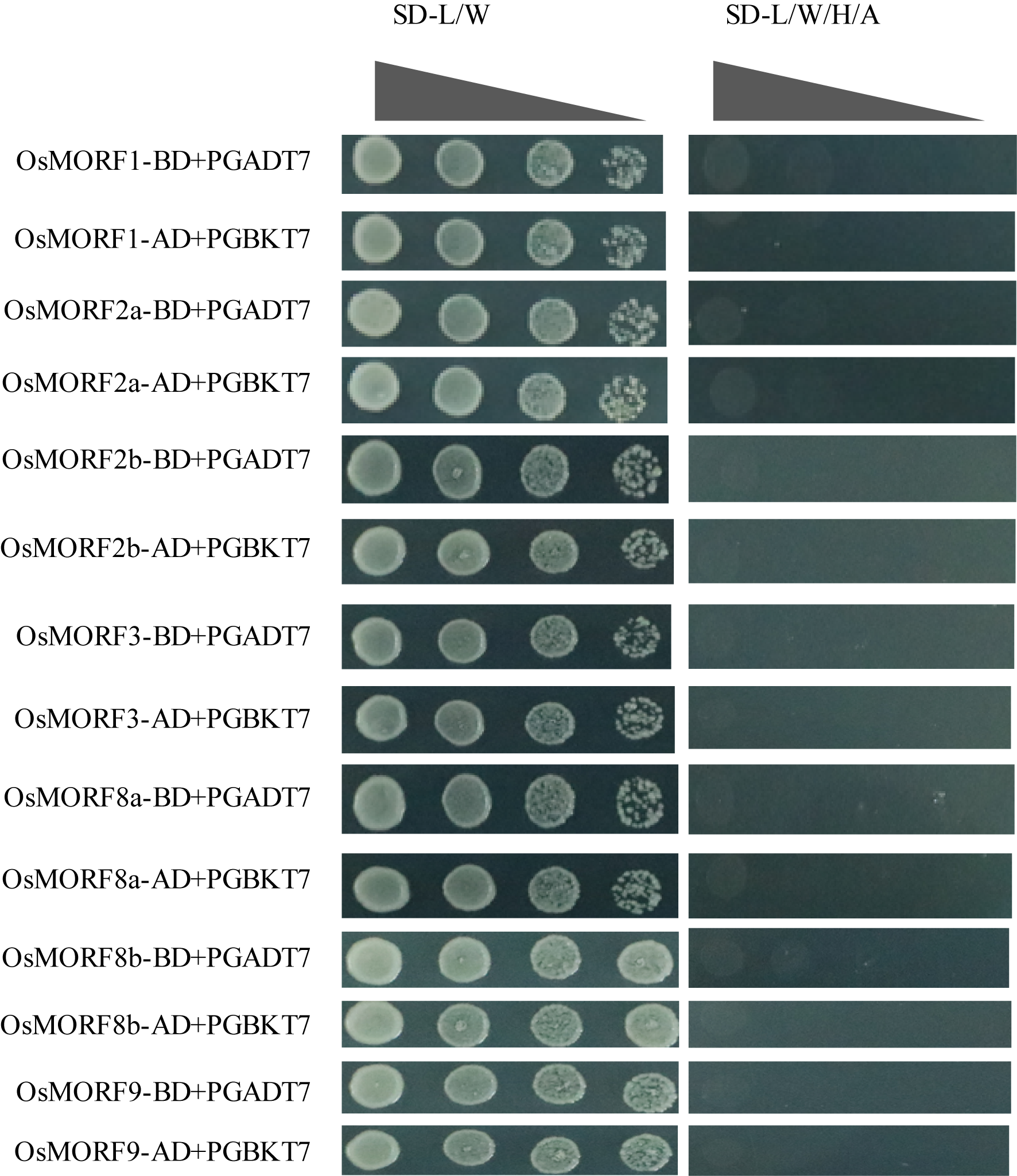

Supplement: Supplementary file 1 [file genes-10-00694-s001.zip › genes-576238- supplemantary/supplementary materials/FigureS2.tif]

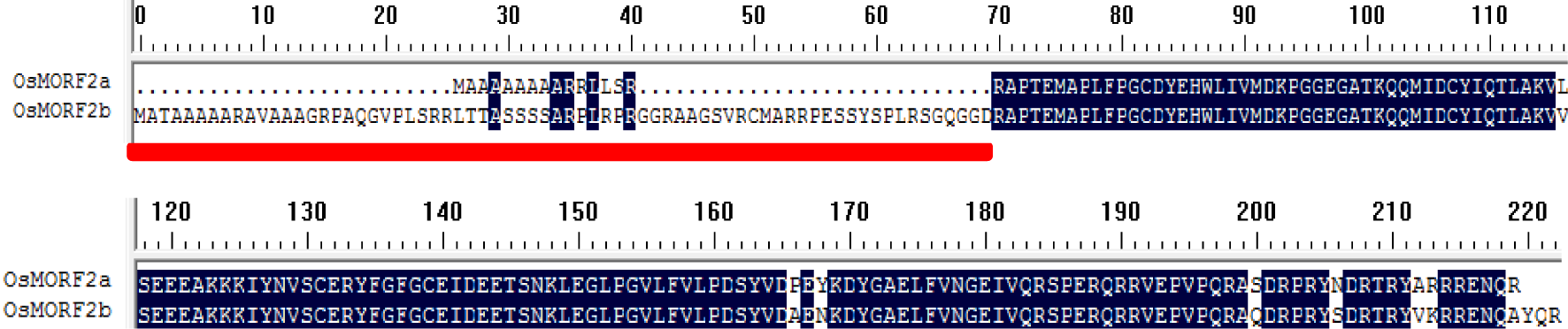

Supplement: Supplementary file 1 [file genes-10-00694-s001.zip › genes-576238- supplemantary/supplementary materials/FigureS3.tif]
